# Pollinator response to livestock grazing: implications for rangeland conservation in sagebrush ecosystems

**DOI:** 10.1093/jisesa/ieae069

**Published:** 2024-08-10

**Authors:** Hayes B Goosey, Gabrielle E Blanchette, David E Naugle

**Affiliations:** Department of Animal and Range Sciences, Montana State University, Bozeman, MT, USA; SWCA Environmental Consultants, Salt Lake City, UT, USA; Wildlife Biology Program, University of Montana, Missoula, MT, USA

**Keywords:** abundance, grazing, livestock, pollinators, rangelands

## Abstract

World food supplies rely on pollination, making this plant–animal relationship a highly valued ecosystem service. Bees pollinate flowering plants in rangelands that constitute up to half of global terrestrial vegetation. Livestock grazing is the most widespread rangeland use and can affect insect pollinators through herbivory. We examined management effects on bee abundance and other insect pollinators on grazed and idle sagebrush rangelands in central Montana, USA. From 2016 to 2018, we sampled pollinators on lands enrolled in rest-rotation grazing, unenrolled grazing lands, and geographically separate idle lands without grazing for over a decade. Bare ground covered twice as much area (15% vs. 7) with half the litter (12% vs. 24) on grazed than idle regardless of enrollment. Bee pollinators were 2–3 times more prevalent in grazed than idle in 2016–2017. In 2018, bees were similar among grazed and idled during an unseasonably wet and cool summer that depressed pollinator catches; captures of secondary pollinators was similar among treatments 2 of 3 study years. Ground-nesting bees (94.6% of total bee abundance) were driven by periodic grazing that maintained bare ground and kept litter accumulations in check. In contrast, idle provided fewer nesting opportunities for bees that were mostly solitary, ground-nesting genera requiring unvegetated spaces for reproduction. Managed lands supported higher bee abundance that evolved with bison grazing on the eastern edge of the sagebrush ecosystem. Our findings suggest that periodic disturbance may enhance pollinator habitat, and that rangelands may benefit from periodic grazing by livestock.

## Introduction

Up to half a trillion USD in global food supplies rely on pollination (Mekouar 2016) making this plant–animal relationship one of the world’s most valued ecosystem services (Klein et al. 2007, Kremen et al. 2007). Insects pollinate more than 80% of the world’s approximate 300 commercial crops (see Allsopp et al. 2008) and rangeland flowering plants (Ollerton et al. 2011), while bees (native and non-native) pollinate approximately 75% of the fruits, nuts, and vegetables grown in the United States (Moisset and Buchmann 2011). Worldwide declines in insect pollinator populations are the result of multiple stressors including habitat loss when native landscapes are converted to row crops, toxicity to pesticides associated with row crops, climate change, and disease (Cunningham 2000, Kremen et al. 2007, Winfree et al. 2009, Koh et al., 2016). The FAO (2016) suggests pollinator conservation as one of the most effective ways to boost food security and support sustainable agriculture.

Rangelands, defined as uncultivated land dominated by grasses, forbs, shrubs, and bare ground, cover a quarter of the earth’s land surface (Alkemade et al. 2013), provide goods and services to over 2 billion people (Sala et al. 2017), and support half of global livestock populations (Havstad et al. 2009). Within the continental United States, 35% of the total land area is rangeland, of which 95% occur in states west of the Mississippi river (Robinson et al. 2019). Primary production on US rangelands is largely driven by annual precipitation, which generally decreases from east to west (Easterling et al. 2017). In the Great Plains, nearly all (91%) rangelands are privately owned, while about half (56%) are in the public domain farther west in the sagebrush ecosystem (Supplemental Fig. 1A–C).

**Fig. 1.**
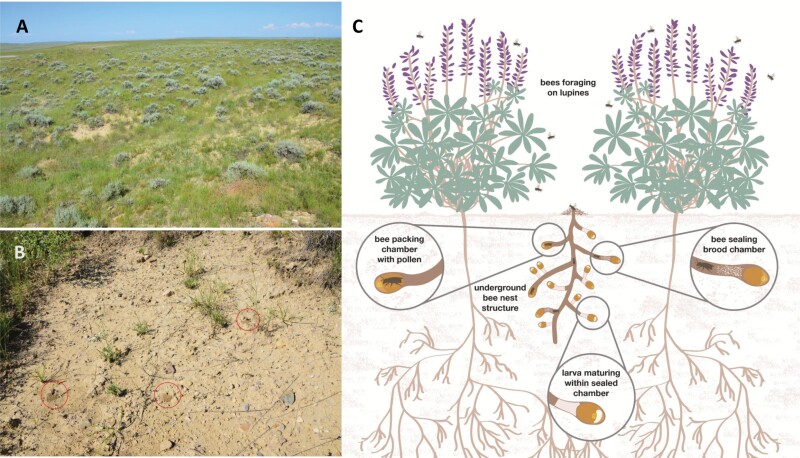
A) Bare ground-nesting habitat in sagebrush steppe rangeland; B) eye-level view of multiple nests; and C) underground schematic of bee nest (photos credits: Hayes Goosey, Montana State University).

Bees are primary pollinators in rangeland ecosystems worldwide (Gilgert and Vaughan 2011), spreading pollen as they forage for nectar from flowering plants (Fig. 1; Pywell et al. 2006, Klein et al. 2007, Isaacs et al. 2009). In US rangeland ecosystems, the availability of native floral resources is a primary determinant of the composition and abundance of bees and other pollinators (Potts et al. 2003, Gilgert and Vaughan 2011, Tuell et al. 2014). Approximately 4,000 different bee species aid in pollination in the United States (Black et al. 2011, Gilgert and Vaughan 2011).

The availability and spacing of bare ground between plants and the depth of dead and decaying litter can also influence pollinator diversity (Fig. 1A; Pei et al. 2023). Bare ground is a natural component of western rangelands and a habitat requisite for 70% of solitary ground-nesting bee species that construct elaborate underground burrows (Fig. 1B) in which they lay eggs and reproduce (Fig. 1C; Black et al. 2011). These bare soils supply suitable substrates into which females excavate their nests (Gilgert and Vaughan 2011) and contribute to soil health (Christmann 2022). Many secondary pollinators such as moths and butterflies, wasps, flies, and beetles also contribute to distributing pollen despite being less efficient than bees (Larson et al. 2018). Collectively, pollinators often connect trophic levels by serving as food items for imperiled grassland songbirds (Maher 1979), gamebirds (Gregg and Crawford 2009, Sullins et al. 2018), and megafauna (e.g., grizzly bears; Gilgert and Vaughan 2011).

Livestock grazing is the most widespread rangeland use (Coppock et al. 2009) and can affect insect pollinators directly or indirectly through herbivory (Cock et al. 2011, 2012). Excessive grazing can directly reduce pollinators by limiting vegetative resources (Gibson et al. 1992, Black et al. 2011), which also supports the intermediate disturbance hypothesis, where pollinator and plant spp. diversity are greatest with moderate disturbance (Rambo and Faeth 1999, Vulliamy et al. 2006, Yan et al. 2014). Moreover, periodic grazing may indirectly promote pollinator diversity and abundance by keeping competitively dominant species in check (Price 1997). Thus, incorporating temporal grazing strategies, which vary seasonal grazing intensity for the purpose of habitat stabilization, may be effective for maintaining arthropod biodiversity in managed rangelands (Goosey et al. 2019).

Properly managed grazing has been shown to improve pollinator resources and increase native bee diversity and abundance (e.g., Vulliamy et al. 2006, Enri et al. 2017). A variety of grazing systems (i.e., seasonal, continuous, rest-rotation) have been studied as methods of range management (Holechek 1981), with rest-rotation grazing (Hormay 1956) most often used for conservation purposes (Sayre et al. 2012, Enri et al. 2017). Rest-rotation grazing is designed to mimic natural patterns of wild ungulate herbivory by moving herds through multiple pastures during a grazing season while resting one pasture to promote plant recovery (Budd and Thorpe 2009, Briske et al. 2011). Morris et al. (1991) proposed rest-rotation grazing of temperate grasslands to support greater pollinator diversity, compared to nonrested lands, by creating more heterogeneous vegetation structure. Similarly, Mitchell et al. (2023) suggest that high-quality bee habitat can be created using targeted grazing by rotating livestock distributions on rangelands, based on flowering plant phenology, to minimize grazing impacts on bee and plant communities. Such was not the case in northern Great Plains grasslands where bee and butterfly abundances were similar among rotational, season-long, and patch-burn grazing treatments (Kral-O’Brien 2022), where patch-burn grazing deploys a fire regime to create a vegetative mosaic on rangelands, which facilitates more even livestock distributions. Despite theoretical benefits, less empirical evidence exists to support rest-rotation grazing as a management tool for enhancing rangeland arthropods in US rangelands (Dennis et al. 1997, but see Enri et al. 2017, Goosey et al. 2019).

To examine effects of rangeland management, we measured the relative abundance, as a proxy of true abundance: hereafter abundance, of bees and other secondary pollinators (i.e., Lepidoptera, Diptera: Syrphidae) on grazed and idle sagebrush rangelands in central Montana, USA. We hypothesized that pollinator abundance would be greater on lands that incorporated livestock grazing due to greater floral resources and higher levels of bare ground resulting in greater food and nesting resources. We further surmised that declines in bee pollinators would coincide with loss of bare ground on idle lands where livestock had been removed for more than a decade. We also thought that abundance of secondary pollinators would decline precipitously with the loss of floral resources on idle versus grazed lands. Lastly, we hypothesized that rest-rotational grazing would have minimal effects on abundance of bee or secondary pollinators.

## Materials and Methods

### Study Area

Research was conducted in Golden Valley and Musselshell Counties, Montana, USA (46.7421N, 108.7854W), comprising mixed private and public land ownerships (Woodward et al. 2011). Ranching families manage private lands for livestock production, and interspersed public lands are managed under multiple-use mandates by the Bureau of Land Management or the Montana Department of Natural Resources. Ranching families often hold grazing leases on adjacent public lands.

Our study area was in rolling terrain between 975 and 1,275 m, with daily temperatures during our sampling period ranging between 2.9 °C and 30.9 °C; average annual precipitation is 359 mm. The vegetation community is classed as intermountain basin big sagebrush steppe (NatureServe 2024), comprising Wyoming big sagebrush, *Artemisia tridentata* spp. *wyomingensis* (Nutt., Asterales: Asteraceae), silver sagebrush, *Artemisia cana* (Pursh, Asterales: Asteraceae), and a variety of perennial rhizomatous and caespitose grasses, including blue bunch wheatgrass, *Pseudoroegneria spicata* (Pursh, Poales: Poaceae), western wheatgrass, *Elymus smithii* (Rydb. and Gould, Poales: Poaceae), green needlegrass, *Nassella viridula* (Trin., Poales: Poaceae), needle-and-thread grass, *Hesperostipa comata* (Trin. and Rupr., Poales: Poaceae), and blue grama grass, *Bouteloua gracilis* (Kunth, Poales: Poaceae). Livestock agriculture is the dominant land use where grazing occurs on native rangelands, while ~10% of land has been converted to row crop, primarily wheat, production.

### Design

Our pollinator study is part of a long-term study designed to assess the effects of grazing on the sage-grouse demographics (Smith et al. 2018a, 2018b) and community structure of ground-dwelling arthropods (Goosey et al. 2019). This study quantified pollinator abundance and *Genus* composition within pastures assigned to 1 of 3 land management treatments (Fig. 1). “Enrolled” pastures are part of the USDA-Natural Resources and Conservation Services’ (NRCS) Sage Grouse Initiative (SGI) launched in 2010 as a voluntary and incentive-based approach for landowners to enroll their property. Enrolled properties operated under individual grazing plans that adhered to the NRCS Montana Prescribed Grazing conservation practice standards. These standards included: (1) livestock use of key forage species ≤50% of the current year’s growth, (2) grazing duration ≤45 days, (3) a yearly change in grazing initiation date by >20 days, and (4) a contingency plan for drought or fire. The NRCS offered an additional monetary incentive for plans that included periodic rest from livestock grazing. These “Enrolled” pastures are rested from grazing for 15 months and provided ≥5% shrub cover, a basic habitat need for nesting sage-grouse. Enrolled pastures were sampled during the latter half of the 15-month rest phase. Other landowners chose not to enroll their property in the SGI, and instead implemented their own grazing systems with unknown rest criteria. To minimize bias, these “Non-enrolled” lands were sampled concurrent with enrolled pastures and also when no livestock were grazing. No livestock grazing occurred in non-enrolled pastures from 1 Jan through the end of our sampling in each study year. Lastly, “Non-grazed” (hereafter “Idle”) pastures were located within the northernmost unit of 3 federal allotments, designated in 1941 as the Lake Mason National Wildlife Refuge (LMWR). The 2,136-ha LMWR has excluded grazing by domestic livestock for over a decade. All enrolled, non-enrolled, and idle sampling locations were separated by a minimum of 0.8 km each of 2016–2018 field seasons.

### Sampling

**Fig. 2.**
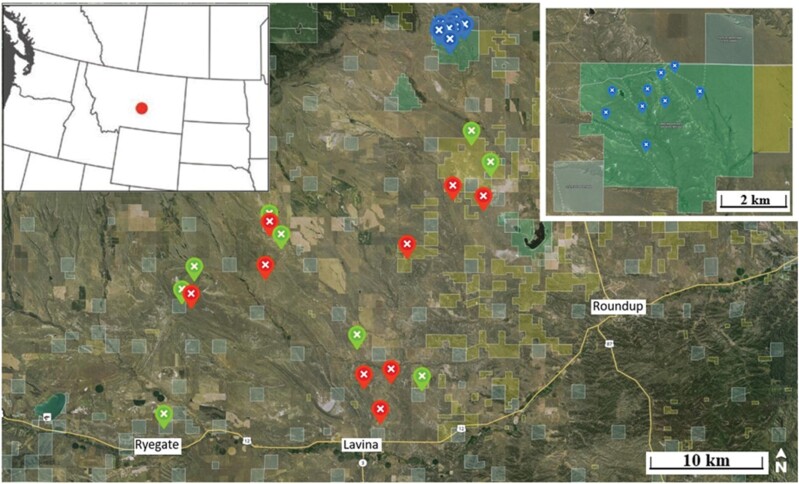
Pollinator and vegetation sampling locations (2016–2018) in enrolled (red), non-enrolled (green), and idle (blue and inset) pastures near Lavina, Montana, USA.

We sampled site characteristics and pollinators in 3 pastures of each treatment (i.e., Enrolled, Non-enrolled, and Idle) each year. New pastures were selected annually, for a total of 27 pastures during this 3-yr study. Each year, one pollinator sampling location was established at the approximate center of each enrolled and non-enrolled pasture to maximize the probability that pollinators we captured were selecting these sites based on available resources rather than incidental captures associated with a pasture edge effect. Three random locations were established on idle LMNR lands (Fig. 2).

### Site Characteristics

Vegetation, bare ground, and plant litter were estimated with ocular sampling once at each site on 9 Jun in 2016 and 5 times at each new site (mid-May through Jun) in subsequent years. Percent cover data was estimated by placing a 20 × 50 cm frame (*n *= 5) 3 m apart on a random bearing transect originating at and extending away from the center of the sampling site (Daubenmire 1959). Vegetation cover was classed as percent flowering forb, nonflowering forb, shrub, grass, lichen, and prickly pear. Percent cover was also estimated for residual plant litter and bare ground.

### Pollinators

We obtained pollinator counts, from mid-May through Jun or early Jul, depending on study year, with 9 traps per site that were spaced 3 m apart along a linear transect determined by a random compass bearing (Droege et al. 2010). Pollinator sampling occurred weekly 5 times in 2016 and 2018 and 7 times in 2017. We employed cup traps as a common, cost-effective, and time-efficient method (Leong and Thorp, 2001, Westphal et al. 2008) with low collector bias (Saunders and Luck 2013). To attract pollinators, traps were colored inside and out, and the blue, yellow, and white appearance fit with surrounding floral resources. Each trap was a 9-cm diameter, 0.5-liter plastic cup with 10% of its base dug into the ground for stability. Each cup was filled two-thirds full of a killing and preservation solution that was 90% water, 9.9% propylene glycol, and 0.1% unscented dish soap. We used this solution to decrease surface tension aiding in submersion and death of landing pollinators (Droege 2015). Weekly, trap contents were collected into 15.24 × 23 cm bags (Whirl-Pak, Nasco Inc., Fort Atkinson, WI, USA), the killing/preservation solution was replenished, and samples were returned to Montana State University for processing. Voucher specimens are placed in the Montana Entomology Collection at Montana State University. We left traps open between weekly collections and kept them visible by clipping vegetation in a 15 cm radius during the growing season.

### Sample Processing

We followed guidelines for cleaning and processing bees as described in *The Handy Bee Manual* (Droege 2015). Generally, specimens were transferred to a fine mesh soil screen, rinsed with cool water to remove large debris, and returned to the collection Whirl-Paks with 95% ETOH for preservation. We then transferred specimens to a petri dish where Order Lepidoptera and Family Syrphidae specimens were identified and counted. Branched hair Order Hymenoptera specimens were removed, labeled with collection metrics, and frozen to −17 °C. Specimens were cleaned in a 473 ml-Ball mason jar with 1 drop of unscented Dawn dish soap, and 29 ml of water. The jar top was secured, shaken for about 60 s, blotted dry, then returned to the mason jar with the top now secured with fine mesh and dried using a commercial Conair hair dryer. Finally, bees were pinned, labeled, and stored in airtight specimen drawers until identified to *Genus* utilizing *The Bee Genera of North and Central America* (Michener et al. 1994).

### Weather and Degree Days

**Fig. 3.**
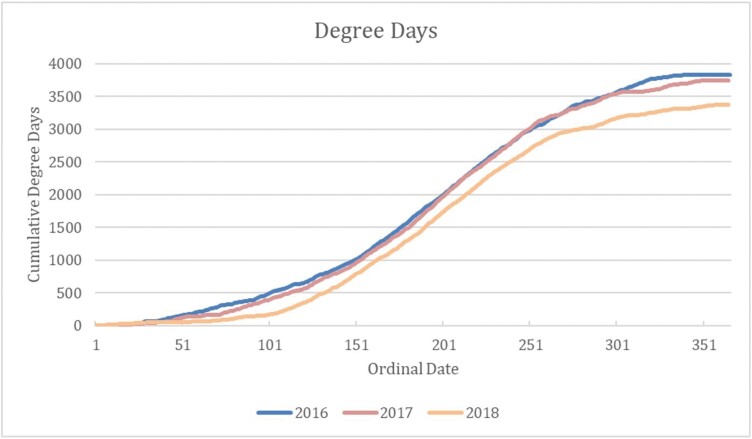
Cumulative degree days (DD) for the Lavina, Montana, USA, landscape calculated from the Horsethief weather station. Pollinator sampling began on 159 DD in 2016, 139 DD in 2017, and 136 DD in 2018.

Wind, temperature, and precipitation data were retrieved from the Horsethief, Montana, HORM8 RAWS weather station at 46.4256 and −108.6742 (Coop 2002; Uspest.org). We supplemented this data set with online NOAA Climate Data from nearby Roundup, Montana (15 SW GHCND: USC00247220; US Climate Data, 2019) because 30-yr averages were unavailable for Horsethief (Table 1). We used degree days as measures of how cold or warm our study area was each year. A degree day compares the average of the high and low temperatures recorded for a location to a standard temperature. Degree days were calculated by selecting the Horsethief, MT HORM8 RAWS weather station and setting the lower threshold to 0 °C and the upper to 30 °C using the single sine method (Coop 2002; Fig. 3).

**Table 1.** Marginal means of site characteristic measures (%) on grazed and idled rangelands (2016–2018) in central Montana, USA, where letters denote statistically different treatments (*α* = 0.05) with all comparison *df* = 2,6. The absence of letters indicates no statistical differences between treatments for that category

	Bare ground	Litter	Shrub	Nonflowering forb	Flowering forb	Lichen	Prickly pear	Grass
Enrolled	16.10a	13.49b	15.35ab	5.88	6.27	6.11	2.78	10.75
Non-enrolled	14.92a	12.93b	20.34a	5.68	6.78	6.13	2.64	8.18
Idle	7.83b	23.77a	13.26b	6.77	6.11	6.39	2.56	8.51
SE	2.64	3.86	2.18	1.95	1.20	1.88	0.16	1.36
*F*-value	5.76	4.96	5.55	0.47	0.17	0.03	0.90	2.11
*P*-value	0.04	0.05	0.04	0.65	0.85	0.97	0.46	0.20

### Statistical Analysis

Twenty-seven pastures were sampled over 3 yr of study, generating 2 data sets: site characteristics and pollinator counts. Each data set was separately subjected to the Proc GLM procedures of SAS (SAS 2016) to test for a year × treatment interaction to determine if data could be combined across years for analyses. If an interaction was detected (*P *< 0.05), yearly data were not combined for analyses. This process resulted in site characteristic data being combined across years and pollinator data analyzed by year.

Site characteristic data were entered by replicate for each collection date as percent coverage. Pollinator counts (abundance) were summed weekly for each replicate into 2 groups of: (1) Hymenoptera pollinators (Hymenoptera [Apoidea]) and (2) secondary pollinators (Diptera [Syrphidae] and Lepidoptera) and data were entered as weekly counts for each pollinator group.

Site characteristics data were normally distributed. Hymenoptera and secondary pollinator data sets failed tests of normality and were over-dispersed with a grand variance 18.6 times greater than the mean. For each data set, selection between linear (GLM) and linear mixed (GLMM) models was determined using small Akaike Information Criterion values estimated from normal, Poisson, and negative binomial distributions run on both transformed and untransformed data. This process resulted in treatment effects being estimated for both pollinator groups on untransformed count data fit to a negative binomial and site characteristics on untransformed percent data fit to a normal distribution within the generalized linear mixed model with treatment as the independent variable and pasture as the random variable. All models included both treatment and replicate as class variables to standardize denominator degrees of freedom across sampling year. A few pollinator traps went missing between collection periods, so weekly count data were standardized using an offset to account for a variable number of traps with the marginal likelihood approximated using Laplace’s method. Marginal means were calculated using the LSMEANS statement of SAS v. 9.4 with differences calculated using the least significant difference (LSD) test (Proc GLIMMIX, SAS 2016).

## Results

### Site Characteristics

Bare ground covered twice as much area (15% vs. 7) with half the litter accumulation (12% vs. 24) on grazed than idle lands regardless of enrollment while grazed lands were similar (Table 1). Shrub cover was 5%–7% greater on grazed lands compared to idle (Table 1). Cover of flowering forbs, nonflowering forbs, grasses, lichen, and prickly pear was similar regardless of treatment (Table 1).

### Weather and Degree Days

Weather parameters and degree days both suggest that 2018 was an unseasonably wet and cool summer that depressed pollinator catches (Tables 2 and 3). Annual precipitation for our study area in 2018 was 42% higher than the 30-yr average (362 mm; 1981–2010). In 2018, daily temperatures were 17%–23% lower than the 2 preceding years. Lower degree days (Fig. 3) likely depressed rate of development and ultimately pollinator catch in 2018.

**Table 2.** Total seasonal counts of primary pollinators, Hymenoptera *Genus* (*subgenus*), and secondary pollinators, Diptera family Syrphidae and order Lepidoptera, for sampling seasons 2016, 2017, and 2018 near Lavina, Montana, USA

				2016			2017			2018		
Genus (subgenus)	Ground-nesting	Pollinator	Cuckoo bee	Enrolled	Non-enrolled	Idle	Enrolled	Non-enrolled	Idle	Enrolled	Non-enrolled	Idle
*Lasioglossum (Dialictus)* ^a^	X	X	X	263	221	53	1,058	749	236	204	107	68
*Agapostemon*	X	X		165	164	72	847	916	361	145	181	95
*Eucera*	X	X		123	71	11	723	497	133	82	78	121
*Halictus*	X	X		35	55	14	117	47	48	22	25	53
*Macrotera*	X	X		30	0	7	0	0	0	0	0	0
*Anthophora*	X	X		22	33	11	122	133	64	38	76	26
*Andrena*	X	X		12	11	3	180	338	68	107	114	94
*Bombus* (excluding *Psithyrus*^b^)		X		10	14	21	51	51	95	23	18	64
*Diadasia*	X	X		10	7	8	7	4	1	0	0	0
*Osmia*		X		5	15	11	28	28	17	17	7	23
*Anthidium*		X		4	2	3	3	3	0	0	0	0
*Panurginu*	X	X		4	0	6	7	1	0	0	0	0
*Lasioglossum (s.str)*	X	X		4	9	9	133	36	146	27	30	57
*Lasioglossum (Sphecodogastra)*	X	X		2	6	2	43	42	48	26	16	23
*Megachile*	X	X		2	1	3	14	4	2	0	0	0
*Melissodes*	X	X		1	1	0	1	0	0	0	1	0
*Lasioglossum (Evylaeus)*	X	X		1	6	14	17	4	12	7	3	9
*Dufourea*	X	X		1	0	3	0	1	2	0	0	0
*Apis*		X		0	1	10	2	5	3	0	8	8
*Nomada*			X	0	0	2	6	7	8	2	3	3
*Ashmeadiella*		X		0	0	1	0	0	0	0	0	0
*Coelioxys*			X	0	1	1	0	0	0	0	0	0
*Hoplitis*		X		0	0	1	0	0	0	0	2	0
*Calliopsis*	X	X		0	1	0	0	0	0	0	0	0
*Colletes*	X	X		0	1	2	6	1	3	0	0	0
*Hylaeus*				0	0	5	0	0	0	0	0	0
*Stelis*			X	0	0	1	0	1	1	0	0	0
*Protandrena*	X	X		0	0	0	0	2	1	0	0	0
*Sphecodes*			X	0	0	0	3	0	1	1	2	1
*Ceratina*		X		0	0	0	1	0	0	0	0	0
Total Hymenoptera				694	620	274	3,369	2,870	1,250	701	671	645
Diptera: Syrphidae		X		3	7	64	52	26	37	7	7	9
Lepidoptera		X		36	39	96	602	302	734	79	102	103
Total secondary				39	46	160	654	328	771	86	109	112
Grand total				733	666	434	4,023	3,198	2,021	787	780	757

^a^ *Dialictus* is comprised mostly of ground-nesting pollinators with a few species exhibiting kleptoparasitism.

^b^Cuckoo bumblebees, zero captured in this study.

**Table 3.** Marginal means of Hymenoptera and secondary pollinator abundance (2016–2018) in rangelands north of Lavina, Montana, USA. Letters denote differences in treatment least squared means (*α* = 0.05) where all comparison *df* = 2,6. The absence of letters indicates no statistical differences between treatments for that category

	2016		2017		2018	
	Hymenoptera	Secondary	Hymenoptera	Secondary	Hymenoptera	Secondary
Enrolled	47.26a	−5.54	151.33a	22.23a	39.13	−1.76
Non-enrolled	60.06a	−3.89	127.86a	6.71b	37.40	0.07
Idle	25.37b	−3.81	50.52b	27.71a	34.40	−1.13
SE	12.32	1.26	12.98	3.74	7.29	1.70
*F*-value	5.85	1.26	25.76	5.96	4.28	0.85
*P*-value	0.04	0.35	<0.01	0.04	0.07	0.47

### Pollinators

#### Bees

We collected 11,094 bee specimens from 27 different genera (Table 2). Specimens from 27, 24, and 16 different bee genera were captured during 2016, 2017, and 2018, respectively. Over all years, *Lasioglossum (Dialictus)*, *Agapostemon*, and *Eucera* were the most common genera captured constituting more than half (58%) of bee specimens. *Halictus* was the fourth most common genera, adding another 7% to the total bee capture. Eleven genera were represented by <10 captures apiece across years (Table 2) including 4 genera (*Ashmeadiella*, *Coelioxys*, *Calliopsis*, *Hylaeus*) that were only present in 2016. For rare genera captured ≤5 times, 2 (*Calliopsis* and *Ceratina*) were only in grazed pastures and 2 others (*Hylaeus* and *Ashmeadiella*) were from idled-only sites. A fifth genera (*Macrotera*) that was also only present in 2016 (*n* = 37 captures) is rarely found as far north as eastern Montana.

#### Secondary Pollinators

We collected 2,305 secondary pollinator specimens (Table 2). In 2016, secondary pollinators were ~8% of total pollinator catch. Lepidopterans were 10-fold more abundant than Syrphidae as secondary pollinators across all years (Table 2). Secondary pollinators were 19 and 13% of the total catch in 2017 and 2018, respectively (Table 2).

#### Treatments

**Fig. 4.**
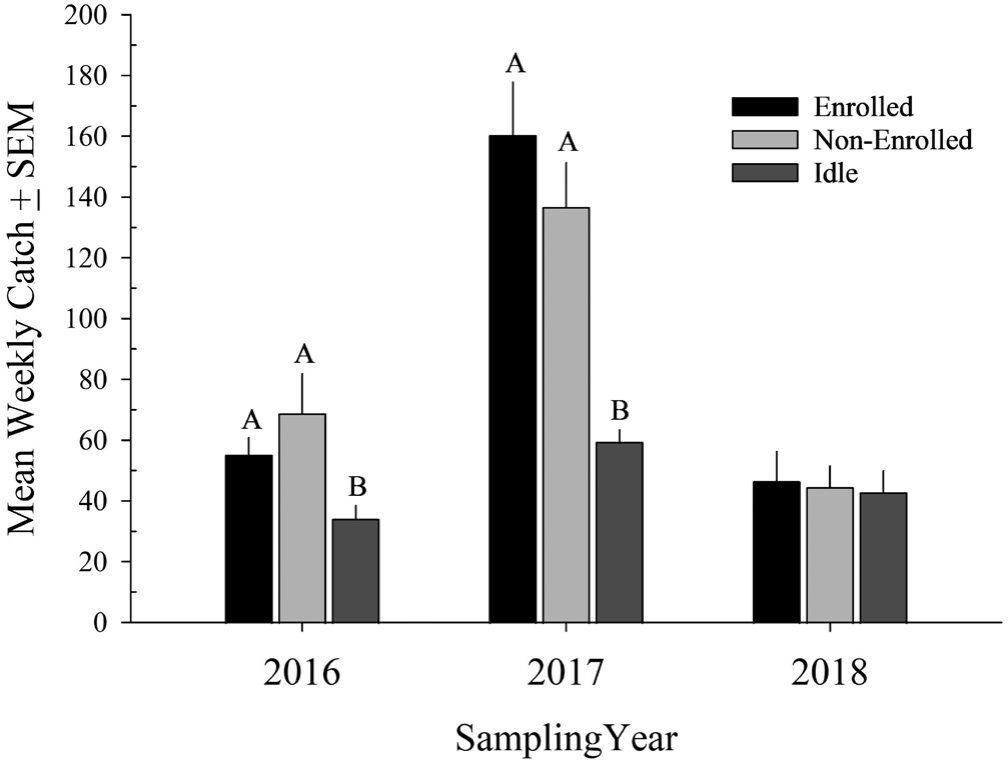
Enrolled, non-enrolled, and idle pasture bee (Hymenoptera: Apoidea) abundance where bars represent weekly marginal means and error bars represent the standard error. Enrolled and non-enrolled pastures are associated with livestock grazing, while livestock has not grazed idle land in over a decade. Sampling was conducted during the 2016–2018 field seasons north of Lavina, Montana, USA. Bars with letters differ (*α* = 0.05); a generalized linear mixed model with random pasture and a negative binomial distribution was fit to count data offset by the number of active pollinator traps; LSD (Proc GLIMMIX, SAS Institute 2008) where all comparisons *df* = 2,6.

In total, we collected 13,339 pollinator specimens (Table 2). Bee pollinator counts (abundance) varied by year within grazing treatments and were 2–3 times more prevalent in grazed than idle pastures in 2016 (*F* = 5.85; *df* = 2,6; *P* = 0.04) and 2017 (*F* = 25.76; *df* = 2,6; *P* < 0.01) (Table 3, Fig. 4). In 2018, bee abundances were similar (*F* = 4.28; *df* = 2,6; *P* = 0.07; Table 3, Fig. 4) among grazed and idled lands during an unseasonably wet and cool summer that depressed pollinator catches region-wide. Abundance of secondary pollinators was similar among treatments in 2016 and 2018 but fewer were captured in non-enrolled pastures during 2017 (Table 3).

## Discussion

A 2- to 3-fold increase in bee abundance in 2016 and 2017 suggested that periodic grazing associated with enrolled and non-enrolled pastures provided suitable nesting habitat for these rangeland pollinators. Vegetation and catch data supported our hypothesis that bee abundance would be higher in grazed pastures where periodic disturbance maintained bare ground and kept litter accumulations in check (Table 3; Fig. 4). Forage consumption and hoof action likely created the unvegetated space required for reproduction by these mostly solitary, ground-nesting bees (Table 3; Fig. 1). In contrast, dense vegetation may have limited pollinator nesting on idled lands with only half the availability of bare ground (7% vs. 15) and twice the litter accumulations (24% vs. 12) as their grazed counterparts. Contrary to our nectar hypothesis, vegetative data implied that flowering plants were not a limiting resource regardless of whether grazing occurred (Table 1). Abundances of secondary pollinators (i.e., butterflies and hover flies) were unrelated to grazing during 2 of the 3 study years (Table 3).

Moderately disturbed sites often house more native bees than undisturbed locations (Michener 2007), invoking the intermediate disturbance hypothesis as a plausible explanation for higher bee abundance with periodic grazing (Lázaro et al. 2016, also see Kati et al 2012, Newbold et al. 2014). Grazing lands in this study supported 9 of the 10 most abundant genera, including the 3 most common groups of ground-nesting bees (Table 2); only the bumble bee (*Bombus*) was more common in idled pastures and may reflect their preference of sites with more vegetative thatch for nest construction (Pugesek and Crone 2021) and their greater flight mobility (Greenleaf et al. 2007) when acquiring floral resources (Dramstad 1996). In these same Montana rangelands, grazing also fostered a higher diversity of ground-dwelling arthropods (Goosey et al. 2019). Meanwhile, on idled lands, detritivore abundance tripled under a simplified community structure that was dominated by predatory Lycosid spiders. Higher arthropod diversity with grazing had trophic implications with arthropods serving as bird-food items for the imperiled greater sage-grouse (Goosey et al. 2019).

Idling large swaths of rangelands could be detrimental to bee populations because most ground-nesting species exhibit breeding-site fidelity, with multiple generations returning to nest in the same pasture (Michener 2007). These life history traits match those of the 2 most abundant communal, ground-nesting bees captured in this study (*Lasioglossum* [*Dialictus*] and *Agapostemon*; Abrams and Eickwort 1981, Wilson and Carril 2016, O’brien and Arathi 2021). Eucera, the third most common bee *genera*, also is a ground-nesting bee that was abundant everywhere, but more so in grazed than idled pastures (Table 2). The benefits of grazing to nesting bees may outweigh the direct impacts of trampling nest sites and compacting soils (Sugden 1985, Gess and Gess 1993, Potts et al. 2005, Vulliamy et al. 2006, Williams et al. 2010). Higher bee diversity in idled pastures would refute this inference, but rarity (captured ≤5 times) had little to no influence with 2 genera detected only in grazed pastures and 2 others from idled-only sites (*see Results*). Changes in seasonal timing of grazing could be made if impacts to genera become apparent in future evaluations (Kimoto et al. 2012).

Periodic grazing that maintains local availability of suitable nesting sites may also help accommodate the limited mobility of many native bees (Black et al. 2011, Wilson and Carril 2016). Most smaller bees move ≤200 m to forage even though some larger species (e.g., bumble bees) can fly >2 km (Michener 2007). *Lasioglossum*, for example, the most abundant genera in this study, are small sweat bees with short flight distances (Waddington and Holden 1979) that are not sensitive to livestock grazing (Kimoto et al. 2012). They suggest that this genus may not be sensitive to grazing due to their limited flight distances to adjacent fields with potentially more resources. In contrast, *Agapostemon* are another common native bee to Northern America that are much larger than *Lasioglossum* and thus capable of longer flight distances to retrieve floral food resources. Members of the *Agapostemon* attempt to maximize their floral rewards to flight energy costs by direct flights to denser floral patches (Waddington and Holden 1979).

The year effect we observed was undoubtedly the result of an unseasonably cool (−20% from average) and rainy (+42% deviation) summer that negated observed differences attributable to grazing in 2016 and 2017. Precipitation and temperature have long been known to affect native bees by impeding their time spent foraging and by influencing the phenological stages of flowering food resources (Lawson and Rands 2019). Such was the case in 2018 when bee densities were similar on grazed and idled lands during an unseasonably wet and cool summer that depressed pollinator catch for bees and other secondary pollinators.

The slightly higher elevational position of idled pastures (~75 m) at the northern edge of our study site (Fig. 2) could have influenced the amount of time bees spent foraging. Similarity in floral resources suggests that this disparity in study design was unlikely to account for the 2- to 3-fold difference observed between grazing and idle treatments. We also did not actually count bee nests but instead inferred from higher densities that grazed pastures better met their reproductive needs. We opted to catch pollinators in colored pan traps instead of emergence traps that have lower capture rates and are time-intensive to set (Cope et al. 2019; but see Sardiñas and Kremen 2014). We too were fearful that emergence traps can under- or overestimate the abundance of communal species of ground-nesting bees (Danforth et al. 1996) depending on trap placement. Of special note, our color pan trapping of the genus *Macrotera* represents a northeast range expansion into central Montana and was our fifth most collected native bee within grazed pastures in 2016 (Table 2). Containing 30 known and mostly specialist species, *Macrotera* is a ground-nesting genera found commonly in the southwest United States and up the Pacific coast (Wilson and Carril 2016).

Rangelands throughout the Great Plains, including central Montana, evolved with bison grazing (Mack and Thompson 1982, Perryman et al. 2021), and our findings suggest that cattle are a suitable surrogate to maintain the requisite disturbance dynamics for ground-nesting pollinators (Enri et al. 2017). Insect biodiversity is an ecosystem service supported globally by cattle grazing and in our case by ground-nesting bees, with far-reaching implications to human food production (Smith et al. 2022).

Grazing provides the one underlying and highly compatible land use that economically sustains rural ranching communities throughout the western United States. More lucrative but less compatible grazing land uses include housing subdivision, cultivation, and other alternatives that in turn fragment remaining public rangelands (Runge et al. 2019, Tack et al. 2019, Bedrosian et al. 2024). Grazing in these comingled landscapes, where private ranchlands are embedded within public rangelands, provides the habitat that sustains large-scale pollinator connectivity (Vasiliev and Greenwood 2023) and wildlife migrations (Tack et al. 2019, 2023).

Policy interventions that restrict grazing on public lands increase habitat loss on private lands and reduce community support for conservation (Runge et al. 2019). Alternately, policy that manages resources on public lands while also supporting sustainable, economically viable ranching operations on private lands is a promising approach for maintaining pollinator habitat. As caveats, we agree that the bumble bee, however, is an exception and livestock grazing programs should consider leaving non-grazed areas, on a rotational basis, to provide necessary bumble bee nesting substrates (Black et al. 2011, Kimoto et al. 2012). In our study, a 15-month rest was applied to enrolled pastures; however, given that bee abundance was similar between grazing treatments, that length of rest seems unnecessary for bumble bee conservation. Rather, a rest of one growing season, from spring through late summer, on select pastures would provide the necessary floral resources for bumble bees while reducing high-intensity disturbances to the land during peak flowering season (Jun–Jul) is likely to benefit pollinator habitat as a whole (Enri et al. 2017). Land managers can further benefit pollinators by deploying livestock grazing as a tool that manages for the inherent heterogeneity of rangelands, which support insect diversity (Öckinger and Smith 2007, Delaney et al. 2015).

## Supplementary Material

ieae069_suppl_Supplemental_Figure_S1
