# Supplementary figures and images for: Pollinator response to livestock grazing: implications for rangeland conservation in sagebrush ecosystems
Source: J Insect Sci. 2024 Aug 10;24(4):13. doi: 10.1093/jisesa/ieae069 (PMC11316144; doi:10.1093/jisesa/ieae069)

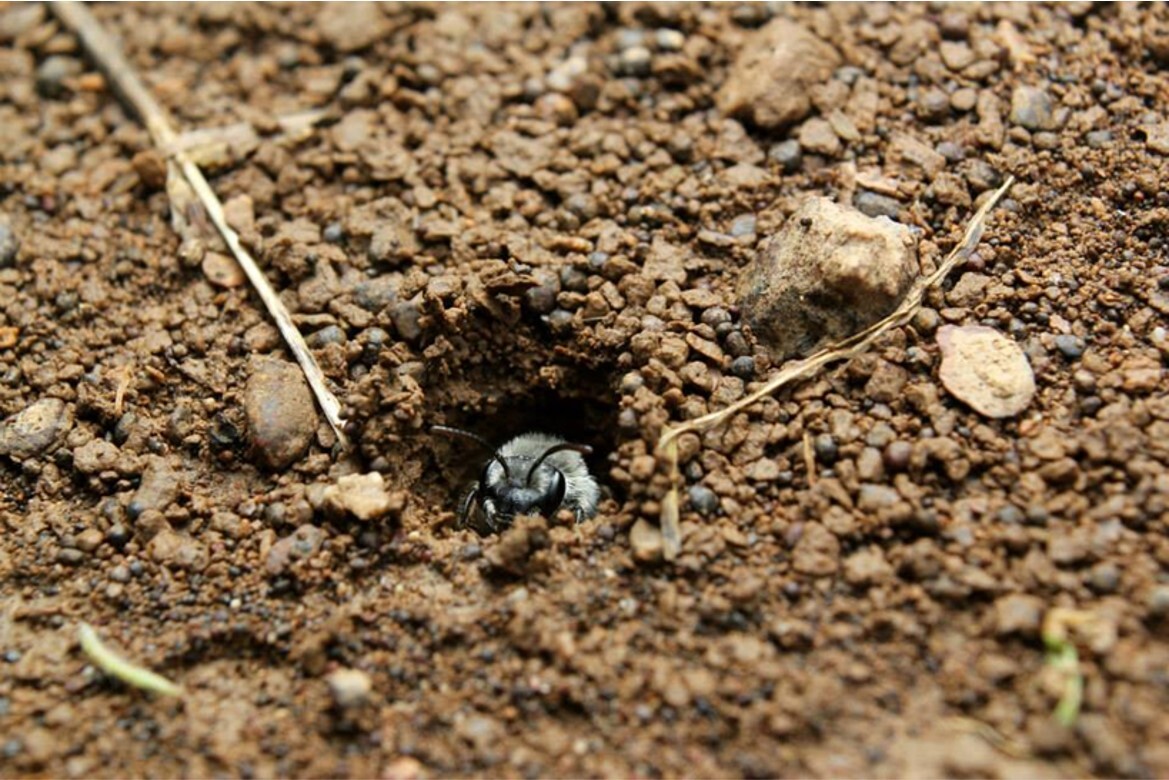

Supplement: ieae069_suppl_Supplemental_Figure_S1 [file ieae069_suppl_supplemental_figure_s1.zip › Supplemental Fig 1C 300dpi.jpg]

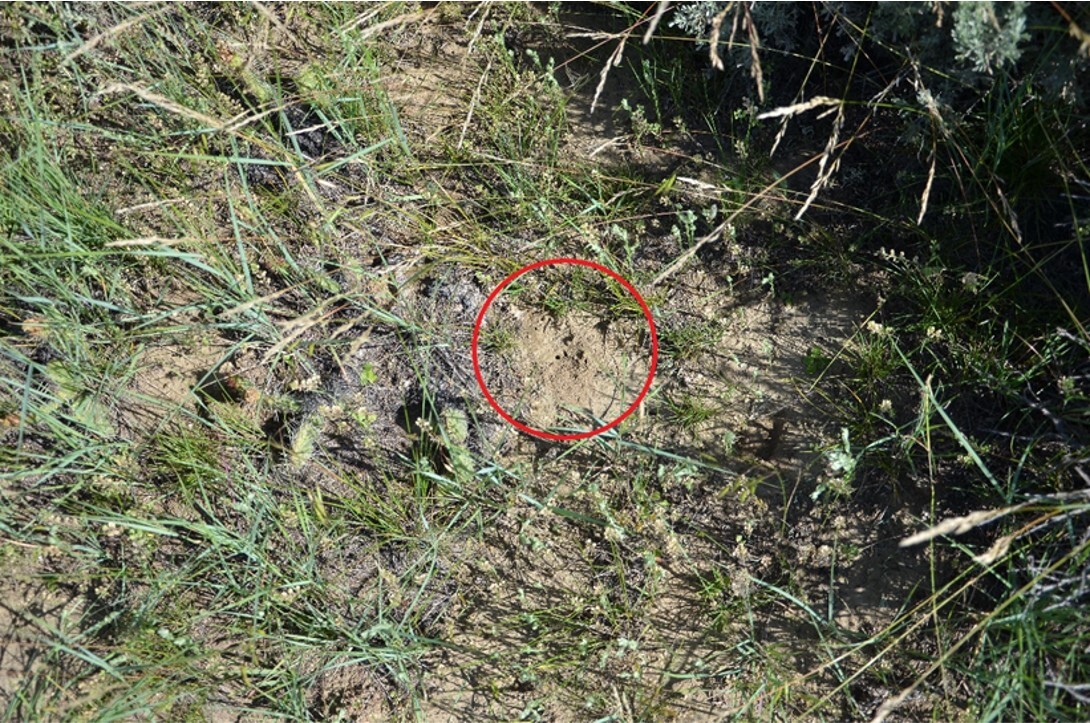

Supplement: ieae069_suppl_Supplemental_Figure_S1 [file ieae069_suppl_supplemental_figure_s1.zip › Supplemental Fig 1A 300dpi.jpg]

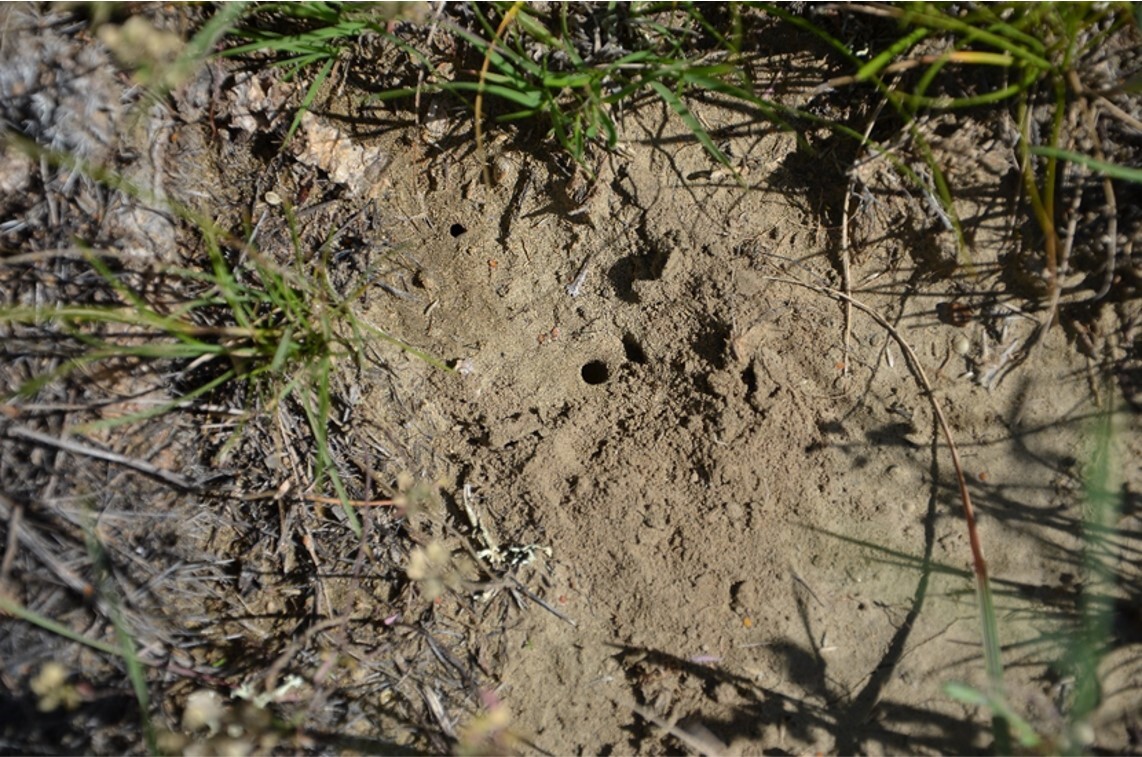

Supplement: ieae069_suppl_Supplemental_Figure_S1 [file ieae069_suppl_supplemental_figure_s1.zip › Supplemental Fig 1B 300dpi.jpg]
